# Evaluation of Short Videos Supporting Healthy Eating and Physical Activity in Early Childhood Education: The Small Bites for Big Steps Pilot Randomised Controlled Trial

**DOI:** 10.1002/hpja.70208

**Published:** 2026-06-18

**Authors:** Rachel A. Jones, Sarah Ryan, Nicole Tate, Ruth Crowe, Arlita Willman, Megan Hammersley

**Affiliations:** ^1^ School of Education, Early Start University of Wollongong Wollongong Australia; ^2^ School of Social Sciences, Early Start University of Wollongong Wollongong Australia; ^3^ Northern Sydney Local Health District Population Health Promotion Brookvale Community Health Centre Sydney Australia; ^4^ School of Medical, Indigenous and Health Sciences University of Wollongong Wollongong Australia

**Keywords:** early childhood, educators, healthy eating, intervention, physical activity, videos

## Abstract

**Issue Addressed:**

Early childhood education and care (ECEC) centres are an ideal setting to promote key healthy eating and active living (HEAL) practices. Redesigning messages around such practices to be digital could help engage ECEC educators and assist with long‐term implementation and reach. The aim of this study was to evaluate the potential impact of a HEAL‐focused video initiative (Small Bites for Big Steps) on precursors to behaviour change including educators' self‐efficacy, perceived behavioural control and behavioural intentions and the acceptability of the videos.

**Method:**

A pilot randomised controlled trial was conducted with early childhood educators (56% aged 25–44 years, 98% female). Participants randomised to the intervention group received on average 3–4 weekly videos promoting HEAL practices, whilst participants in the control group maintained usual practice. Educators' self‐efficacy, perceived behavioural control, and behavioural intention were assessed using questionnaires at baseline and post‐intervention (9 weeks). Data were analysed using Mann Whitney *U* tests in SPSS and thematic analysis. Acceptability data were collected using questionnaires, focus groups, interviews, and Vimeo analytics.

**Results:**

One hundred and six educators from 16 ECEC services were recruited. Exploratory efficacy analysis found significant improvements from baseline to post‐intervention between groups for healthy eating/drinking perceived behavioural control. At post‐intervention, there was a significant difference between intervention and control groups for healthy eating/drinking behavioural intention, physical activity behavioural intention, and overall behavioural intention. No significant between group differences were observed for changes in perceived behavioural control relating to physical activity or for any self‐efficacy measures. Educators valued the content, length and approach of the videos. Limitations identified from the qualitative data included technical difficulties and cultural appropriateness.

**Conclusions:**

This video‐based intervention positively influenced educators' perceived behavioural control and behavioural intentions, key precursors to behaviour change. The video suite was acceptable to educators.

**So What?:**

Redesigning HEAL messages into video resources may support ECEC educators to promote and implement these practices to young children and their families/carers.

## 1 Introduction

The eating patterns and physical activity levels of Australian children, including young children (birth to 5 years) are suboptimal. Currently only 19% of 2–4‐year‐old Australian children meet the dietary guidelines for vegetable intake [1], with children in this age group consuming 30% of their energy from discretionary foods [2]. Only 6% of 3‐year‐old Australian children are meeting the guidelines for physical activity (which includes total 180 min per day of physical activity and 60 min per day of energetic play) [3].

Eating and physical activity patterns track from early childhood to childhood and from childhood to adolescence and adulthood [4, 5, 6]. Therefore, establishing healthy eating and physical activity patterns in early childhood is critical. Several different approaches have been trialled to optimise young children's healthy eating and physical activity. These approaches have targeted different settings (e.g., homes, early childhood education and care (ECEC) settings and public spaces and have been diverse in nature, content and delivery mode (online and face to face)) [7, 8, 9, 10]. Although such approaches can be feasible and acceptable and have reported favourable changes in children's healthy eating and physical activity outcomes [7, 8, 9, 10], their reach and potential sustainability is typically limited. Sustainable programs that reach whole communities are urgently needed to ensure optimal healthy eating and physical activity patterns for all young children [11]. Programs delivered in collaboration with State Government Health or Education Departments may be a feasible option for extended reach and ongoing sustainable implementation.

Within the Australian context, New South Wales (NSW) Health recognises the need for early and effective promotion of healthy eating and physical activity and hence one of their key strategic priority areas is Healthy Eating and Activity Living (HEAL) [12]. Over the past 15 years they have funded several programs to promote HEAL practices, one of which includes *Munch & Move*. *Munch & Move* is a free program available to all NSW registered ECEC services (preschools, long day care services and family day care services) and provides evidence‐based training and resources to promote healthy eating and physical activity for children aged birth to 5 years [12]. Implementation of the program by ECEC educators is supported by NSW Health's Local Health District (LHD) health promotion officers [13]. Health promotion officers are responsible for implementing health promotion initiatives within local health districts. During the COVID pandemic, educators' engagement with *Munch & Move* declined due to a rapid change is service priorities [11]. In response to this, LHDs needed to explore new and innovative ways to re‐engage educators as the need for HEAL messages remained high, as do programs that have extended reach and long‐term sustainability [11]. The NSLHD Early Years Team recognised this decline and examined the needs of ECEC educators participating in the *Munch & Move* program. Formative research (unpublished) highlighted ECEC educators were seeking new, diverse and engaging resources, for example short videos (in addition to what is already available) including additional contextual information that extended on the *Munch & Move* key messages and those that were delivered by allied health professionals to assist with *Munch & Move* implementation. Videos are a favoured format for ECEC educators to receive training [14] and previous interventions utilising videos have been found to be effective in modifying school‐aged children's healthy eating and physical activity behaviours [15, 16, 17, 18].

As a result of this formative research the NSLHD Early Years team developed a suite of short videos (ranging in duration from 1 to 3 min) which focused around and extended upon the key HEAL *Munch & Move* messages. The content was delivered by NSLHD health professionals including, Health Promotion Officers, a Child & Family Health Nurse and a variety of child, youth & family allied health professionals. The video content was informed by national guidelines and recommendations and covered developmental milestones for children birth to 5 years old. All scripts were developed by the NSLHD Early Years Team and content reviewed by an expert advisory group including NSLHD Allied Health professionals, ECEC educators and researchers from Early Start, University of Wollongong. Content was also carefully developed to align with the NSLHD culturally diverse population. To strengthen clarity and engagement, script development was guided by the Telethon Kids Institute's *How to Tell the Core Story of Early Childhood* short guide (https://www.thekids.org.au/projects/HPER/core‐story/), which draws on framing theory to communicate early childhood messages effectively. This approach informed language choices and tone, ensuing messages were acceptable and relevant to educators. The videos aimed to address common barriers identified by educators, such as translating guidelines into everyday practice and engaging families, providing practical, age‐specific extensions of existing *Munch & Move* resources. A key priority for the NSLHD Early Years team was evaluating the acceptability and feasibility of the videos prior to future studies which investigate educator/child behaviour change and implementation fidelity and in time wide‐spread dissemination. The team were also interested in exploring the potential impact of the videos on educators' self‐efficacy, perceived behavioural control and behavioural intention. Self‐efficacy, perceived behavioural control and behavioural intention are precursors to behaviour change and have been successfully assessed in other ECEC‐targeted studies (e.g., Bruijns et al. [19]). Therefore, the aim of this study was to explore the potential impact of the videos on these intended outcomes and to evaluate the acceptability of the Small Bites video suite.

## 2 Methods

The evaluation was approved by the University of Wollongong Human Research Ethics Committee (2021/ETH11784). Participants were recruited between March and May 2022 and data were collected from May to October 2022.

To evaluate the potential impact of the videos a small pilot randomised controlled trial was conducted. *Munch & Move* trained ECEC services (*n* = 420) and their educators located within NSLHD were eligible and invited to participate in the evaluation. This equated to approximately 90% of the ECEC services within the local health district. A formal sample size calculation was not performed due to the pilot nature of this trial, which aimed to assess acceptability and explore the potential impact of the videos, rather than efficacy. Rather, the sample was determined pragmatically, based on the number of eligible ECEC services within NSLHD that had previously completed Munch & Move training.

Services were recruited through ECEC service emails which were available on the ACECQA website, *Munch & Move* communications delivered by NSLHD and phone calls (using the service numbers). Staff from UOW and NSLHD facilitated recruitment. Interested services were sent information sheets about the evaluation and then individual educators provided online consent. All educators from the recruited services, irrespective of experience, were invited to participate in the evaluation. No other inclusion and exclusion criteria were applied. Recruited services were randomised in a staggered 1:1 ratio (using a computerised random number generator) by a data manager with no other involvement in the evaluation to either the intervention group or a control group. Participants were not blind to group allocation.

Educators in the intervention group then received the suite of videos via text messages. Text messaging is a highly accessible and low‐cost communication method that has been used in the delivery of a range of health promotion intervention studies, with promising outcomes [20]. Videos were stored and made accessible to educators through the Vimeo online platform. Between April and August 2022, educators in the intervention group were sent on average 3–4 secure Vimeo links via text message each week, for 8–9 weeks. The selection of videos received by each educator was dependent on the age group that they currently worked with (0–18 months, 0–3 years, 18 months‐3 years, 2–5 years or 3–5 years—as indicated in the baseline questionnaire), with most age groups receiving videos for 8 weeks, and educators working with children aged 0–3 years receiving videos for 9 weeks. Some videos were specific to a particular age group, while some were consistent across all age groups. To ensure relevance and avoid overwhelming educators, only videos related to the children they were responsible for were shared. An example of the sequencing of videos sent to educators is shown in Supporting Information S1. Educators were asked to watch each video and engage in the content of the video. During the intervention period, both the intervention group and the control group had access to NSW Health Department *Munch & Move* online resource library as per usual.

### 2.1 Potential Efficacy

Following randomisation, educators were emailed a link to the baseline questionnaire. The baseline questionnaire was completed online using REDCap. The questionnaire included demographic questions and questions about educator's self‐efficacy and perceived behavioural control. Questions pertaining to self‐efficacy focused on healthy eating/drinking, physical activity, and communication with parents and questions pertaining to perceived behavioural control focused on healthy eating/drinking and physical activity. Educators were asked to indicate on a scale of 0 (strongly disagree) to 10 (strongly agree) their response to questions. Example questions included: ‘On a scale from 0 (not confident at all) to 10 (completely confident) how confident are you in your ability to perform the following during work: [1] transitioning children from using a bottle to cup, [2] encouraging children to drink water, [3] providing children with regular active play opportunities irrespective of the weather, [4] providing opportunities for children to participate in tummy time and [5] talking with families about their children's health eating behaviours’. Questions were sourced from valid and reliable questionnaires [21, 22]. The questionnaire took approximately 15 min to complete. Harms were assessed nonsystematically (i.e., educators were asked to contact the researchers if they had concerns about the research).

In relation to potential impact, the primary outcomes for this study were changes in educator's self‐efficacy, perceived behavioural control and behavioural intention. Quantitative data were analysed using appropriate statistical analyses completed in SPSS (IBM Corp. Released 2022. IBM SPSS Statistics for Windows, Version 29.0. Armonk, NY: IBM Corp). Complete case data analysis was performed. Demographic and acceptability data were analysed using descriptive statistics. Baseline and post‐intervention data (9 weeks post baseline) were analysed using non‐parametric tests due to the distribution of the data. Change scores (baseline to follow‐up) were computed for self‐efficacy (healthy eating and drinking, physical activity, overall), perceived behavioural control (healthy eating and drinking, physical activity and overall). Mann Whitney U tests were used to compare these change scores between intervention and control groups. Between group differences in behavioural intention (healthy eating/drinks, physical activity and overall (both healthy eating/drinks and physical activity)) were only assessed at post‐intervention. Between‐group post‐intervention differences for behavioural intention were analysed using Mann–Whitney U tests. An alpha level of 0.05 was set as the threshold for statistical significance for all analyses.

All educators (including those from the intervention group and the control group) were invited to participate in a post‐intervention questionnaire at the end of the intervention period (9 weeks post baseline). This questionnaire was similar to the baseline questionnaire but also included nine additional questions which focused on educators' behavioural intentions (0 = strongly disagree to 10 = strongly agree). Examples of the behavioural intention questions included ‘I am going to encourage children to choose water as their main drink’, ‘I am going to provide more time for all children to develop their locomotor skills’. Questions assessing behaviour intentions were also were sourced from valid and reliable questionnaires [21, 22]. The post‐intervention questionnaire took approximately 20 min to complete. At the completion of the post‐intervention questionnaire, educators were offered the opportunity to enter a draw to win one of two $200 gift cards as a gesture of thanks for their time.

### 2.2 Acceptability

Qualitative data were collected to explore components of acceptability (including engagement).

At the end of the intervention period, educators in the intervention group were also invited to participate in an online focus group or a one‐on‐one online semi‐structured interview. These were facilitated by researchers from the University of Wollongong (RJ/MH, females with PhD) and a research and engagement organisation to dive deeper into educators' acceptability of the videos. The researchers did not have previous relationships with the participants. Focus groups and one‐on‐one interviewers, guided by a structured interview guide, lasted approximately 20 min. Questions about self‐efficacy, perceived behavioural control and behaviour intention in relation to HEAL practices were asked. Examples of questions asked included ‘What were the most useful parts of the video series?’, ‘What were the least useful parts of the video series?’, ‘Were there any barriers to using the videos and the content?’, ‘How has your knowledge on healthy eating changed?’ All participants were compensated for their time with a $50 gift card. Focus groups and interviews were audio recorded and transcribed verbatim. Participants were provided with an opportunity to view the transcripts prior to analysis.

Video acceptability and engagement was assessed using questionnaires, Vimeo analytic data and through the interviews and focus groups. Each week after accessing each video, educators were provided with a link to two questions to ascertain their acceptability of the videos. The first question focused on the perceived usefulness of the video (1 = extremely useful to 5 = not at all useful), and the second on whether the videos had given them ideas on how they could change their practice (Yes/No/Unsure). These questions took approximately 30 s to complete. Vimeo analytic data, which include the number of views, were collected throughout the intervention period. Educators discussed their thoughts pertaining to video acceptability and engagement in the interviews and focus groups (as described above).

Qualitative data were analysed manually using thematic analyses [23]. The data were deidentified and then deductively analysed by two researchers (RJ, MH). Topics were discussed between the researchers to ensure consensus. Data from the focus groups and interviews were triangulated with the questionnaire data [23]. The researchers involved in the qualitative analysis (RJ, MH) have extensive experience in qualitative methods and extensive experience conducting research with early childhood educators.

The methods used for the quantitative component were mapped against the CONSORT 2025 Expanded Checklist (Supporting Information S2). The methods used in the qualitative case studies were mapped against the Consolidated Criteria for Reporting Qualitative Studies (Supporting Information S3).

## 3 Results

**FIGURE 1:**
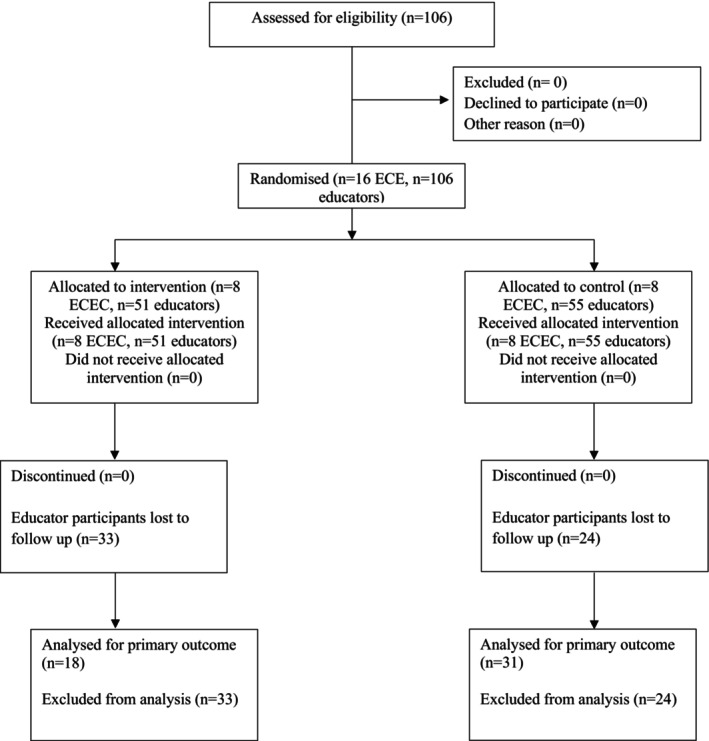
Flow of participants through study.

One hundred and six educators (from 16 ECEC settings) provided consent and completed the baseline questionnaire. Fifty‐one educators were randomised to the intervention group and 55 to the control group. Forty‐nine (45%) educators completed the post‐intervention questionnaire (18 from the intervention group and 31 from the control group). Figure 1 shows the flow of educators through the evaluation. Most educators involved in the evaluation were female (97%), aged between 25 and 44 years (57%) and spoke English (62%). Over 40% of educators involved in the evaluation had worked in sector for over 10 years and nearly 90% had worked in the sector for at least 2 years. Educators' qualifications were fairly evenly split between Certificate III (26%), Diploma (34%), and Bachelor degree (35%). More than half of the educators worked with children aged 3–5 years (53%). Detailed information pertaining to the demographics of the educators involved in the evaluation are shown in Table 1.

**TABLE 1:** Participant demographic characteristics.

Demographics	Intervention group (*n* = 51)	Control group (*n* = 55)	All (*n* = 106)
Age	*n* (%)	*n* (%)	*n* (%)
15–24	5 (9.8)	1 (1.8)	6 (5.7)
25–44	32 (62.7)	28 (50.9)	60 (56.6)
45–64	13 (25.5)	25 (45.5)	38 (35.8)
65 and over	1 (2.0)	1 (1.8)	2 (1.9)

Gender	*n* (%)	*n* (%)	*n* (%)
Female	48 (94.1)	55 (100.0)	103 (97.2)
Male	2 (3.9)	—	2 (1.9)
Both	1 (2.0)	—	1 (0.9)

Language spoken at home	*n* (%)	*n* (%)	*n* (%)
English	32 (62.7)	34 (61.8)	66 (62.3)
Mandarin	5 (9.8)	5 (9.1)	10 (9.4)
Spanish	4 (7.8)	—	4 (3.8)
Korean	5 (9.8)	3 (5.5)	8 (7.5)
Hindi	—	3 (5.5)	3 (2.8)
Cantonese	2 (3.9)	2 (3.6)	4 (3.8)
Other (specify)	3 (5.9)	8 (14.5)	11 (10.4)

Time in early childhood education	*n* (%)	*n* (%)	*n* (%)
< 6 months	4 (7.8)	—	4 (3.8)
6 months—2 years	4 (7.8)	5 (9.1)	9 (8.5)
2 years—5 years	18 (35.3)	11 (20.0)	29 (27.4)
5 years—10 years	7 (13.7)	12 (21.8)	19 (17.9)
> 10 years	18 (35.3)	27 (49.1)	45 (42.5)

Highest qualification	*n* (%)	*n* (%)	*n* (%)
Certificate III	13 (25.5)	14 (25.5)	27 (25.5)
Diploma	19 (37.3)	17 (30.9)	36 (34.0)
Bachelor	17 (33.3)	20 (36.4)	37 (34.9)
Masters	2 (3.9)	4 (7.3)	6 (5.7)

Age group	*n* (%)	*n* (%)	*n* (%)
0–18 months	4 (7.8)	1 (1.8)	5 (4.7)
18 months—3 years	5 (9.8)	7 (12.7)	12 (11.3)
0–3 years	8 (15.7)	4 (7.3)	12 (11.3)
2–5 years	10 (19.6)	11 (20.0)	21 (19.8)
3–5 years	24 (47.1)	32 (58.2)	56 (52.8)

There was a significant difference between the intervention and control groups in the change in perceived behavioural control for healthy eating and drinking from baseline to post‐intervention (U = 130.50, p0.002), with a large effect size as indicated by the rank‐biserial correlation (r(b) = 0.53) (Table 2). No significant between group differences were observed for changes in perceived behavioural control relating to physical activity or for any self‐efficacy measures. For behavioural intention, which was measured only at post‐intervention, there was a significant difference between the intervention and control groups for healthy eating and drinking (U = 177.00, *p* = 0.02), with a medium effect size as indicated by rank‐biserial correlation (r(rb) = 0.37); physical activity (U = 166.50, *p* = 0.01), with a medium effect size as indicated by rank‐biserial correlation (r(rb) = 0.40); and overall behavioural intention (combined healthy eating drinking and physical activity) (U = 160.50, *p* = 0.02), with a medium effect size as indicated by rank‐biserial correlation (r(rb) = 0.42) (Table 2).

**TABLE 2:** Medians and IQR for self‐efficacy, perceived behavioural control and behavioural intention.

Domain	Baseline median (interquartile range)			Post‐intervention median (interquartile range)		
	Control (*n* = 55)	Intervention (*n* = 51)	All (*n* = 106)	Control (*n* = 31)	Intervention (*n* = 18)	All (*n* = 49)
Self‐efficacy						
Healthy eating/drinking	9.83 (1.67)	9.00 (2.25)	9.58 (2.00)	9.67 (1.00)	9.83 (1.00)	9.67 (1.00)^a^
Physical activity	9.00 (2.54)	8.50 (1.77)	8.67 (2.39)	9.00 (2.00)	9.33 (0.78)	9.17 (1.68)
Overall healthy eating and physical activity self‐ efficacy	9.17 (1.96)	8.75 (1.80)	8.93 (1.88)	9.17 (1.33)	9.50 (0.95)	9.36 (1.33)
Healthy eating/drinking communication	8.50 (2.50)	8.50 (3.00)	8.50 (3.00)	8.50 (2.00)	9.50 (1.50)	9.00 (2.00)
Physical activity communication	9.00 (2.00)	8.00 (3.00)	8.00 (3.00)	9.00 (2.00)	9.50 (2.00)	9.00 (2.00)
Overall communication self‐efficacy	8.50 (2.33)	8.33 (3.00)	8.33 (3.00)	8.33 (2.00)	9.50 (1.67)	9.00 (2.00)
Perceived behavioural control						
Healthy eating and drinks	9.67 (2.00)	8.67 (2.00)	9.00 (2.00)	9.33 (2.00)	9.67 (1.33)	9.33 (1.67)
Physical activity	9.00 (2.00)	9.75 (2.00)	9.00 (2.00)	9.00 (1.75)	10.00 (1.00)	9.00 (1.63)
Behavioural intention						
Healthy eating/drinking	—	—	—	9.67 (1.50)	10.00 (0.50)	9.75 (1.00)^a^
Physical activity	—	—	—	9.20 (1.60)	10.00 (0.20)	9.80 (1.10)^a^
Overall	—	—	—	9.43 (1.44)	10.00 (0.39)	9.67 (1.11)^a^

*Note:* All scores were on a scale of 0 to 10 (where 0 denotes not confident at all and 10 denotes completely confident for self‐efficacy and perceived behavioural control questions and 0 denotes strongly disagree and 10 denotes strongly agree for behavioural intention questions).

^a^ Indicates significant difference between intervention and control groups.

Four educators from different ECEC services participated in the interviews/focus groups (two participants attended a focus group and two participants attended individual interviews). These participants had viewed the video series for 2–5 year old (*n* = 1) and 3–5 year old (*n* = 3) children. Perceived strengths and limitations and perceived changes in educators' knowledge and practice and possible recommendations were identified. No harms were reported.

### 3.1 Perceived Strengths and Limitations

Overall, educators suggested that the quality of the videos, the inclusion of real people in real settings doing real work, the length of the videos and the evidence‐based content were strengths. The inclusion of real people and the use of evidence‐based content meant that the information was relatable and contextually appropriate. The brevity (i.e., the length) of the videos enabled educators to watch the videos during the workday which made them more accessible compared to the *Munch & Move* modules. The videos that focused on breastfeeding and water consumption were perceived a particularly useful. Some educators suggested that they had been involved in the *Munch & Move* Program for so long that they had become complacent, and the videos were a good reminder of the key *Munch & Move* messages and why they were important.

And seeing the actual people in the videos demonstrating how—I think that was really instrumental in helping us to change our program as such. It has completely changed our Munch & Move curriculum, the videos—we have the [fact] sheets but seeing people putting them into practice really highlighted the benefits of it too. (FG2)

I thought it just brought us some fresh ideas, I think you get a bit complacent when you've been doing it for many years so some of the ideas we could just tweak and others were like that's a new way of looking at it. So yes it was certainly worthwhile. All of my room leaders did it and I've got 8 rooms across my service and they all found it really useful. (FG1)

The main perceived limitation related to technical difficulties accessing and viewing the videos. These limitations were also mentioned by educators in the post‐intervention questionnaire. The videos on occasions were not compatible with different phones and tablets. Although technical difficulties were experienced, the technical difficulties were always seemingly quickly resolved, however, the technical difficulties did disrupt momentum and engagement.

I just found the accessibility hard sometimes. Like initially when we first got it, it was fine, it worked really well and then it didn't. And that was hard when we kept interested in getting like a new one's uploaded and it was uploaded, but then I couldn't see, but then it rectified itself and it was fine, but I had to play a bit of catch up. So that was the only issue. (Int 2)

Further, there were mixed feelings about the number of videos; some educators suggested that there were too many videos, resulting in a sense of ‘catching up’ all the time, however other educators suggested that delivery of the videos was spaced out well and once a routine was established it was not too overwhelming. Educators also mentioned that the videos offered limited culturally appropriate ideas and given the diversity within the NSLHD communities, the inclusion of a variety of culturally appropriate foods would have been helpful.

### 3.2 Perceived Changes in Educators' Knowledge and Practice

Educators participating in the focus groups and interviews uniformly suggested that their knowledge in the key content areas (e.g., breastfeeding, fundamental movement skills, provision of water and healthy eating opportunities) had increased and on many occasions that resulted in change in pedagogical practice. There were two very specific and clear examples of this. Example 1 related to the video about breastfeeding. This video highlighted the importance of regular breastfeeding, including during the day. The educators had identified that many of their mothers were working and had chosen not to breastfeed during the day. As a result of the breastfeeding video, they were challenged to consider alternatives for their mothers to enable breastfeeding during the day.

lots of working mothers and we have nursery spaces and they tend not to breastfeed during the day for various reasons, so coming at it that from a different angle and talking about the brain development and the benefits of bonding with the child and giving them a few more options to come and breastfeed—most thought they'd just pump during the day, so that was good and a different way of looking at it. It took a bit of negotiating to turn things around—we had an area in the classrooms, but giving them a space to have a breastfeeding room that parents owned, and the parents had input into that room, so that was really nice … we had a breastfeeding space but we didn't have a dedicated room as such and we had a few mothers that came in and used it and it was really nice because it meant we had a bit of a mothers group going on too. (FG1)

Example 2 related to the videos about water consumption. Two educators from different services described the changes that had been implemented because of these videos. As a result of the information contained in the videos, educators encouraged children, through interactive age‐ appropriate challenges, to increase their water intake as well as increasing the educators' own water intake.

With the water video—we actually set up a little challenge with the kids and educators, they had to fill out how much water they had each day—they had to colour in a water droplet for each water bottle they filled, and we had an incentive, and when they drank their quota they got a sticker. So that was really great. And it was good for us too (educators) so we weren't getting left behind (FG2)

Other educators mentioned that their knowledge relating to fundamental movement skills and healthy eating was enhanced. They suggested that the videos prompted them to rethink how children should move and identify some of the specific movement skills needs of the children. Educators' knowledge pertaining to specific movement skills were increased with one educator specifically mentioning that she had learnt how to throw correctly after watching the videos.

Educators also suggested that the videos had resulted in better communication and interactions with the children and their families/carers in relation to the key focus areas. Educators mentioned how they modified their language after watching the videos (e.g., eating a rainbow of foods) which resulted in more meaningful conversations and interactions with the children. Educators also suggested that the content of the videos had provided them with extra confidence to speak to their families/carer community about the key health behaviours mentioned in the videos.

I've started using phrases like ‘eat the rainbow’. It's my new favourite thing to say, instead of doing some paperwork when the kids are eating, I sit with them and I have a little plate of food and talk about what colours we can see, what veges do we have, what colours of the rainbow are missing and what we can add. So that's been really good and I love that it has made me a lot closer with the kids—instead of thinking what can I write about this as an observation, what can I do, like almost having an ulterior motive, instead of just being with them in the moment… (FG2)

### 3.3 Possible Recommendations

Educators mentioned several recommendations that could be considered in future iterations of the videos. These have been summarised in Table 3 with supporting quotes.

**TABLE 3:** Recommendations from interviews and focus groups.

Recommendation	Supporting quotes
1. Text messages containing video links are an appropriate medium to distribute information.	I find texts so much better because they look at them, I know myself I don't have notifications on my emails because I don't want to hear it all the time. (Int 1) I never answer my emails so yes text is the way to go… Yeah I think having the text message notification just left there until you look at it—helps. (FG2) Yeah I think text is better—it's more instantaneous. (FG1)
2. Text messages containing links to the video increases communication between educators	I've just recently set up an educator Whatsapp chat so something like that would be really easy to do I would just flick it [the video link] to the Whatsapp group. (Int 1) Yes I've got all their [educators] numbers. Many are linked up to it [the program] separately but if they did miss it I can just flick it to other educators. (FG1) I love to send out little messages saying this is our helpful tip for the day so the videos I received from you guys has really helped generate content. So redistributing the information has been really valuable. It hasn't just stopped at us it has continued past us, so it might be just two of us here tonight, but we've shared it with lots of people and shared the information. (FG2)
3. Kid friendly versions of the videos would be helpful and potentially well used	I think that those videos would be good if they made some that were kid friendly—to share with them, there aren't really any other ways the kids learn about fundamental movement apart from just doing it, so if they could watch something and understood the concept—and even kids love technology, so if they could have some interactive technology. (FG2)
4. Additional topics could be included in the video suite (e.g., allergies, celebration foods, dental hygiene, different cultural foods in the healthy food choices)	I think they need to talk about celebration foods—we want to celebrate but if we had a cake for every child's birthday we'd have a cake every day, and that would be really hypocritical as we talk about sometimes and everyday foods!—and there is only so much you can do with watermelon, it's a bit boring after a while, so perhaps some different ideas. And a few more different cultural ideas represented in healthy food choices. And a bit more on allergies—what are some of the options for only rice flour, gluten free or even some reliable websites to go to would be of assistance. (FG1) The other thing that I think they could do some more with is dental hygiene—Swish and swallow, especially with Covid, it's hard for young children to swish and swallow without spitting—because spitting is the fun bit—to spit on the table! So more dental (hygiene) for the younger ones would be good for me. (FG1) More on the Swish and Swallow, I don't have much about that. (FG2)

A total of 297 responses were received for the two acceptability questions (perceived usefulness and ideas to change practice) over the intervention period. The response rate varied depending on the age group and focus area (i.e., healthy eating/drinking and physical activity). More responses were received to the questionnaire from educators who received videos pertaining to behaviours in 2–5 year old children compared to younger children (i.e., < 2 years). For example, nine responses were received from educators on videos targeting healthy eating for birth to 3 years, however 101 responses were received from educators on videos targeting healthy eating for 3–5 year old children. Acceptability data were not available for more than 30 videos. Several videos were deemed moderately useful, very useful or extremely useful and only two videos in total were perceived as not being very useful at all.

More than three quarters (76%, 225/297) of the educators who responded to the acceptability questions suggested that the videos had provided ideas for them to change their practice. Less than 10% (9%, 27/297) suggested that the videos had not provided them with ideas for change and 15% (45/297) suggested that they were unsure if the videos had provided them with ideas of how to change their pedagogical practice.

Vimeo analytics allowed the number of ‘plays’ (i.e., the number of times a video was played) to be calculated, which is displayed in Supporting Information S4. The number of ‘plays’ ranged from 1 to 58 times and the plays gradually decreased over the 9‐week period. The introductory video was played 58 times. Two videos (healthy eating learning experiences and stability skills) were played 26 and 21 times respectively. Seventeen videos were played between 11 and 20 times and 29 videos were played 10 or less times. The mean percent played ranged from 0% to 100% and averaged 69%. The mean percent is the percentage of the video that is watched. The five videos that had high plays and mean percentage watched included lunchboxes, encouraging water consumption, oral health for preschoolers, fine motor skills and fundamental movement skills—teaching moments.

## 4 Discussion

This is the first Australian evaluation within the ECEC sector to explore the impact of videos, delivered via text message, on educators' self‐efficacy, perceived behavioural control and behavioural intention in relation to HEAL.

Self‐efficacy is the belief in one's ability to execute behaviours. In this study self‐efficacy was assessed as educator confidence in promoting HEAL messages. It is well documented that educators generally lack self‐efficacy/confidence, for example previous studies have indicated that educators lack confidence in estimating serving sizes [24] and providing meaningful physical activity learning opportunities for children [25]. No changes in self‐efficacy were reported in this study, however educators did mention that their self‐efficacy/confidence had increased in relation to communicating about some of the key HEAL messages with parents/carers. These results contrast with other studies that have shown significant changes in educator's healthy eating related self‐efficacy following targeted interventions [26, 27, 28, 29]. For example, the *Appetite to Play* intervention in Canada, delivered through workshops and e‐learning modules, significantly increased early years educators' knowledge and confidence in implementing healthy eating practices [26]. Similarly, in relation to physical activity studies, three studies from Canada have shown that pre‐service and in‐service educator's self‐efficacy regarding physical activity and sedentary behaviour can be improved significantly following participation in online e‐learning training modules [19, 27, 28]. Modifying self‐efficacy is a complex process that is influenced by several factors including personal and professional experience and employment status (full time vs. part time) [27]. It is plausible to suggest that the small intervention dose and the short exposure to the videos may not have been enough to improve educator's self‐efficacy, however our findings showed some promise that it could. Additionally, it is plausible to suggest a stronger foundation in theory, for example Social Cognitive Theory, may have been beneficial as other studies have attributed changes in educator's self‐efficacy to use of such theories [29]. Further studies utilising videos for a longer exposure time are needed to investigate this further. In contrast to other studies that have assessed educator's self‐efficacy, to the best of our knowledge this is the first study that has provided information via videos and text messaging, thus further work exploring these mediums is recommended.

In this study, a significant difference in changes over time in perceived behavioural control for healthy eating and drinking between the intervention and control groups was identified. Additionally, significant differences of medium effect size were also reported for behavioural intention (healthy eating/drinking, physical activity) and overall (both healthy eating/drinking and physical activity) at post‐intervention. These results align with other studies that report changes in perceived behavioural control and behavioural intention. For example, Bourke et al. [28] conducted a RCT involving 209 ECEC educators. Medium effect sizes were reported for educator's perceived behavioural control and behavioural intention at post intervention (4 weeks). Behavioural control is defined as the amount of control a person has over a situation (i.e., how easy or difficult it is to change behaviours or interest) [30]. Behavioural control theory posits that if a person has the sense of control, they are more likely to be able to change behaviour [30]. Behavioural intention aligns closely with behavioural control and is underpinned by the motivational factors that influence a given behaviour, where the stronger the intention to perform the behaviour, the more likely the behaviour will be performed [30]. It is plausible to suggest that the significant differences in educators perceived behavioural control (healthy eating and drinking) informed the significant changes in behavioural intention (healthy eating and drinking) and in turn possible changes in pedagogical practice, as detailed in this study. The pedagogical changes mentioned in this study included increased fundamental movement skill opportunities. We speculate that the branding of the videos (i.e., branded as an initiative from NSW Health) and the fact that the information in each video was presented by health professionals may have been contributing factors in the change of behavioural control and intention. It is possible that the educators may have felt like they had permission to make changes that related to the video content in their ECEC settings, however further data would be needed to confirm this.

However, it remains difficult to understand why significant differences were observed in healthy eating/drinking and overall behavioural intention, but not in perceived behavioural control and overall perceived behaviour control for healthy eating/drinking. One possible explanation is that food‐related practices in ECEC settings are complex. Educators often have limited involvement in decisions around food service menus and contents of lunchboxes typically provided by parents. While interviews highlighted pedagogical changes, enhanced water consumption and increased breastfeeding opportunities—indicating a shift in educators' intention to promote healthy eating—these changes may not have extended to a stronger sense of control over food provision. The boundaries of educators' capacity to influence in this area may therefore be a barrier to shifts in perceived behavioural control.

It is also possible that the exposure time was too short to impact on perceived behavioural control‐ both in the number of videos and the length of the evaluation period (i.e., 9 weeks). Although this may have been a contributor, it is important to note that the engagement (as reported via video analytics) declined over time, thus if the intervention were longer, strategies would need to be implemented to encourage ongoing engagement with the videos. Furthermore, for both the intervention group and the control group the answers to the baseline questionnaire were fairly high (i.e., median above 8.50 out of 10). That is educators perceived their behavioural control as highly favourable (perhaps more than it was in reality). This is a common phenomenon for questionnaire data and often results in a ceiling effect, meaning there is little room for improvement [31]. Additionally, it is possible that educators involved in the study had a bias to the content area. Educators self‐selected to be involved in the study, and it may have been that the educators that self‐selected had a heightened interest in the focus area (i.e., HEAL messages) and thus already had high self‐efficacy and perceived behavioural control. An increased sample size which captures a broader diverse range of educators is recommended. Trialling the videos with non‐Munch *& Move* trained ECEC services may potentially have a greater effect. Finally, it is difficult to ascertain the full extent of exposure of the videos as on some occasions the videos were watched together (i.e., as a group rather than as individuals). A greater exposure to more educators in more ECEC settings may result in more favourable self‐efficacy and perceived behavioural control intervention outcomes. Further, consistent messaging presented on multiple occasions in multiple scenarios may be likely be more effective than a one‐off viewing. For example, (a) links for videos could be used in regular memos sent out by the LHD and NSW Health, (b) links for the videos could be posted on social media sites (e.g., Facebook), (c) videos could be used in professional development, specifically state‐wide professional development related to *Munch & Move* (both face‐to‐face and e‐learning professional development), (d) Health promotion officers could intentionally talk about the videos during onsite visits, (e) Directors could view and discuss the videos in regular staff meetings, (f) Videos could be promoted state‐side *Munch & Move* conferences (g) nomination of a HEAL champion may ensure that the videos are regularly viewed and actively reflected upon to inform practice and pedagogy.

One of the greatest challenges in this study was the engagement of educators with the videos. Despite the use of an easily accessible platform (i.e., text messages) and regular prompts to engage with the videos, the Vimeo analytic data showed relatively low number of ‘plays’ and engagement with the videos. It is well documented that educators have a number of complex competing responsibilities. These responsibilities have exponentially increased in recent years [25], and even more so, following COVID‐19. Investigating opportunities to increase educator engagement will be important to consider in ongoing studies. Text messaging seems to be a favourable method of dissemination in health promotion interventions [20], but perhaps discussion needs to focus on when the text messages are delivered, and the number of text messages being sent. These are areas to be considered in future iterations.

The results of this evaluation need to be considered in light of the following limitations. First the response rate for the post‐intervention questionnaire was much lower than expected (45% retention rate). Several factors may have contributed to this, including the limited time for educators to complete questionnaires. It is well known that educators are overburdened with administrative tasks and the completion of the questionnaires may have simply been an additional administrative task for educators and did not take priority. Additionally, educators who had engaged in a minimal number of videos over the intervention period may not have felt comfortable completing the questionnaire knowing that their self‐efficacy, behavioural control and intention had not changed. Second, although the questionnaire comprised valid and reliable questions from other questionnaires, the questionnaire, as a whole, was not tested for validity and reliability. Third, all data were self‐reported, which may have resulted in social desirability bias [32] and may not be reflective of actual self‐efficacy, perceived behavioural control and behavioural intention. Future evaluations could consider objective measurements, for example observing educators' practices and impact on child behaviours. Fourth, a limited number of educators participated in the interviews and focus groups despite extensive efforts to recruit educators (including phone calls emails, text messages and incentives), and thus data saturated may not have been reached and the results may not be representative of the wider educator population involved in the study. Data were not collected on those who chose not to participate. Additionally, educators working with birth to 3‐year‐old children were underrepresented. Limited data were collected from this sub‐population. Fifth, baseline data were collected after randomisation, which may introduce a risk of selection bias and allocation of participants to groups based on their knowledge of baseline characteristics. However, all randomisation was completed by the data manager who did not have access to the baseline data, and all baseline data were collection prior to participants receiving the intervention. As such, baseline data reflect preintervention status, although this sequencing is acknowledged as a limitation. Finally, regarding the analysis, we were unable to account for clustering by service due to convergence issues with linear mixed model analyses. Consequently, Mann–Whitney *U* tests were used, which do not adjust for clustering and may underestimate variability. Missing data were handled using complete case analysis, which may have introduced bias and reduced generalisability if data were not missing completely at random.

## 5 Conclusion

Establishing healthy eating and physical activity patterns in early childhood is critical for long‐term health and wellbeing. Reenergising ECEC educators to promote such messages through everyday practices is a key health promotion strategy. Although this study was relatively small in duration and sample size, there were a number of promising findings that could have positive implications for the long‐term promotion and implementation of HEAL messaging within the ECEC setting. Findings suggests repackaging HEAL messages into short, easily accessible videos, has the potential to positively influence ECEC educator confidence and perceived behavioural control. Evidence indicated the use of video footage, filmed within an ECEC setting, may be instrumental in providing ECEC educators with relatable and easily applicable ideas for positive pedagogical change. The HEAL messages continue to be a priority area within the Australian context, as evidenced by the inclusion of such messages in national curricula and provision of national funding. Ensuring the successful promotion of these messages and application of these messages in ECEC will continue to be key in the promotion of healthy eating and physical activity for young children. This study highlights the potential use of low cost and high reach resources to ensure the translation of policy into practice. Further studies which include larger samples sizes and specific strategies to enhance educator's engagement and participation will be important to confirm these results and further the promotion of the HEAL messages through ECEC settings.

## Funding

This study was supported by the Preventative Research Support Program, NSW Health and Northern Sydney Local Health District, Population Health Promotion team.

## Ethics Statement

Ethics approval was granted by the University of Wollongong Human Research Ethics Committee (2021/ETH11784).

## Consent

Participants consented for this research to be published.

## Conflicts of Interest

The authors declare no conflicts of interest.

## Supporting information


**Supporting Information: S1.** Example of video schedule.

**Supporting Information: S2.** CONSORT 2025 Expended Checklist.

**Supporting Information: S3.** Consolidated criteria for reporting qualitative studies.

**Supporting Information: S4.** Number of ‘Plays’ for each video.

## Data Availability

The data that support the findings of this study are available on request from the corresponding author. The data are not publicly available due to privacy or ethical restrictions.
