# Supplementary material for: Evaluation of Short Videos Supporting Healthy Eating and Physical Activity in Early Childhood Education: The Small Bites for Big Steps Pilot Randomised Controlled Trial
Source: Health Promot J Austr. 2026 Jun 18;37(3):e70208. doi: 10.1002/hpja.70208 (PMC13280184; doi:10.1002/hpja.70208)
Supplement: Supplementary file 1 — Supporting Information: S1. Example of video schedule. [file HPJA-37-0-s001.docx]

**Supplementary Material 1: Example of video schedule**

| **Birth to 3 years** | **Week** |
| --- | --- |
| Welcome to Small Bites for Big Steps | 1 |
| Small Bites for Big Steps empowering educators in the first 2000 days | 1 |
| Creating a breast friendly service | 1 |
| Eye tracking | 1 |
| Tummy time | 1 |
| Safe sleep | 2 |
| Swaying and rocking | 2 |
| Tired signs | 2 |
| Strategies for baby not taking a bottle | 2 |
| Rolling | 3 |
| Crawling | 3 |
| Transiting to solids | 3 |
| Settling an infant | 3 |
| Walking | 4 |
| Best foods to start with | 4 |
| Promoting stability skills | 4 |
| Oral health for babies | 4 |
| Appropriate drinks for babies | 5 |
| Promoting physical activity with babies | 5 |
| Oral health for toddlers | 5 |
| Fine motor skills | 5 |
| Promoting locomotor skills | 6 |
| Appropriate drinks for toddlers | 6 |
| Promoting manipulative skills | 6 |
| Encouraging vegetable consumption | 6 |
| Healthy eating learning experiences for 18 months to 3 years | 7 |
| Vestibular system | 7 |
| Encouraging water consumption | 7 |
| Role modelling behaviours | 7 |
| Transitioning to a cup | 8 |
| 24 hour movement guidelines 18 months to 3 years | 8 |
| Fuss free mealtimes | 8 |
| Empowering educators in supporting families | 8 |
| Strength | 9 |
| How to have challenging conversation | 9 |
| 24 hour movement guidelines 0-18 months | 9 |
| What is the blue book? | 9 |
