# Supplementary material for: Evaluation of Short Videos Supporting Healthy Eating and Physical Activity in Early Childhood Education: The Small Bites for Big Steps Pilot Randomised Controlled Trial
Source: Health Promot J Austr. 2026 Jun 18;37(3):e70208. doi: 10.1002/hpja.70208 (PMC13280184; doi:10.1002/hpja.70208)
Supplement: Supplementary file 4 — Supporting Information: S4. Number of ‘Plays’ for each video. [file HPJA-37-0-s004.docx]

**Supplementary Material 4: Number of ‘Plays’ for each video**

| **Video** | **Total number of plays** |
| --- | --- |
| Small Bites for Big Steps: Empowering Educators | 58 |
| Healthy Learning Experiences | 26 |
| Stability Skills | 21 |
| Lunchboxes | 17 |
| Encouraging Water Consumption | 16 |
| Oral Health for Preschoolers | 16 |
| Fundamental Movement Skills - Teaching Moments | 15 |
| Fine Motor Skills | 15 |
| Appropriate Drinks | 15 |
| Fuss Free Mealtimes | 15 |
| Promoting Social Skills through Active Play | 15 |
| Fundamental Movement Skills - Locomotor | 15 |
| Role-Modelling Behaviours | 14 |
| Encouraging Vegetable Consumption | 14 |
| 24 Hour Movement Guidelines | 13 |
| What is the Blue Book? | 12 |
| How to Have Challenging Conversations | 12 |
| Self-Regulation Through Active Play | 12 |
| Empowering Educators in Supporting Families | 11 |
| Indoor Active Play | 11 |
| School Readiness | 10 |
| Creating a Breastfeeding Friendly Service | 10 |
| Fundamental Movement Skills: Manipulative | 10 |
| Eye Tracking | 9 |
| Tummy Time | 7 |
| Swaying and Rocking | 7 |
| Promoting Locomotor Skills | 7 |
| Walking | 7 |
| Safe Sleep | 6 |
| Appropriate Drinks | 6 |
| Rolling | 6 |
| How to Promote Physical Activity for Babies | 6 |
| Vestibular System | 5 |
| Promoting Stability Skills | 5 |
| Strategies for Baby not taking a Bottle | 4 |
| Tired Signs | 4 |
| Transitioning to Solids | 4 |
| Promoting Manipulative Skills | 4 |
| Appropriate Drinks | 4 |
| Healthy Learning Experiences | 4 |
| Oral Health for Toddlers | 3 |
| Best Foods to Start with | 3 |
| Crawling | 3 |
| Transitioning to a Cup | 3 |
| 24 Hour Movement Guidelines | 3 |
| Strength | 2 |
| Oral Health for Babies | 2 |
| 24 Hour Movement Guidelines for Babies | 2 |
| Settling an Infant | 1 |
